# The Relationships of Dentition, Use of Dental Prothesis and Oral Health Problems with Frailty, Disability and Diet Quality: Results from Population-Based Studies of Older Adults from the UK and USA

**DOI:** 10.1007/s12603-023-1951-8

**Published:** 2023-08-24

**Authors:** Rachel Kimble, A.O. Papacosta, L.T. Lennon, P.H. Whincup, R.J. Weyant, J.C. Mathers, S.G. Wannamethee, S.E. Ramsay

**Affiliations:** 1 Population Health Sciences Institute, Newcastle University, NE2 4AX, Newcastle upon Tyne, UK; 2 Division of Sport and Exercise Science, School of Health and Life Sciences, University of the West of Scotland, Blantyre, UK; 3 Department of Primary Care and Population Health, UCL, NW3 2PF, London, UK; 4 Population Health Research Institute, St George's, University of London, Cranmer Terrace, SW17 0RE, London, UK; 5 Department of Dental Public Health, School of Dental Medicine, University of Pittsburgh, Pittsburgh, Pennsylvania, USA; 6 Institute of Health and Society, University of the West of Scotland, Technology Ave, G72 0LH, Blantyre, Glasgow, UK

**Keywords:** Ageing, disability, frailty, oral problems, dentures

## Abstract

**Objectives:**

This study examined the relationships of dental status, use and types of dental prothesis and oral health problems, individually and combined, with diet quality, frailty and disability in two population-based studies of older adults.

**Design:**

Cross-sectional study.

**Setting and Participants:**

Men form the British Regional Heart Study (BRHS) (aged 85±4 years in 2018; n=1013) and Men and Women from the Health, Aging, and Body Composition (HABC) Study (aged 75±3 years in 1998–99; n=1975).

**Measurements:**

Physical and dental examinations and questionnaires were collected with data available for dental status, oral problems related to eating, diet quality, Fried frailty phenotype, disability based on mobility limitations, and activities of daily living (ADL). The associations of dental status and oral health problems, individually and combined, with risk of frailty and disability were quantified. The relationship with diet quality was also assessed.

**Results:**

In the BRHS, but not HABC Study, impaired natural dentition without the use of dentures was associated with frailty independently. This relationship was only established in the same group in those with oral problems (OR=3.24; 95% CI: 1.30–8.03). In the HABC Study, functional dentition with oral health problems was associated with greater risk of frailty (OR=2.21; 95% CI: 1.18–4.15). In both studies those who wore a full or partial denture in one or more jaw who reported oral problems were more likely to have disability. There was no association with diet quality in these groups.

**Conclusion:**

Older adults with impaired dentition even who use dentures who experience self-report oral problems related to eating may be at increased risk of frailty and disability. Further research is needed to establish whether improving oral problems could potentially reduce the occurrence of frailty and disability.

## Introduction

As in most economically developed countries, the number of older adults is increasing rapidly in the UK and USA, with approximately 22–24% of the population estimated to be aged 65 years and over by the year 2050 (1). In addition, life expectancy has increased without a corresponding increase in healthy life years so that many more individuals spend their later years with life-limiting health conditions that impede on their quality of life and independence (2, 3). For example, prevalence of frailty increases whilst disability-free life expectancy decreases markedly beyond the age of 70 years (4, 5). Consequently, frailty-related conditions account for a large proportion of health and social services usage and costs (6, 7, 8). Thus, identifying potentially modifiable factors related to frailty and disability and the development of preventative measures has become an important public health priority (9).

Good dental health is an increasingly recognised factor in healthy ageing (10). Nonetheless, tooth loss increases in prevalence with age; for example, according to the recent Adult Dental Health Survey, approximately 50% of adults aged 75–84 years had functional dentition (commonly defined as ≥21 teeth), compared with just 32% aged 85 or over (11). Our research group has previously shown that older adults with impaired dentition (<21 natural teeth) are more likely to be frail and have mobility limitations compared to those with functional dentition (12, 13). It has been well established that impaired dentition is associated with altered food selection and compromised nutritional status (14, 15) which might represent a potential underlying pathway for the association between frailty and disability (16, 17). More recently we reported that individuals with <21 natural teeth who did not use dentures had higher odds of frailty regardless of denture use (18). However, we couldn't differentiate by denture types due to limited data available for the time point of that study. Nevertheless, the types of dentures (e.g., full or partial) represent different challenges for restoration of masticatory performance (19) and subsequently diet quality (20, 21, 22). Therefore, the expectation is tenable that denture types as well as dentition might be important indicators of frailty and disability, but this has received little attention. In support of this, Everaars and colleagues (23) previously reported that compared to individuals with natural teeth those with partial or full prothesis were 1.5 and 3.3 times more likely to be frail, respectively, however they did not include functional dentition in that study.

We and others have also previously shown that subjective oral health problems that are relatively easy to assess are independently associated with, weakness, frailty and disability in older adults (18, 24, 25). However, the joint associations between dentition, denture use and types combined with oral health problems on frailty and disability has received little attention. Semba et al (26) reported that denture wearing older women, were more likely to be frail among those with self-reported difficulty chewing/swallowing (24%) compared with those without such difficulty (10%). The same authors also found that the denture wearers with difficulty chewing/swallowing had the lowest concentrations of micronutrients in plasma supporting the hypothesis of diet as a potential mediator. Similarly compared to older adults with fixed partial dentures those with poor quality removable dentures (partial and full) were more likely to have reduced mastication and less muscle mass (27). Nonetheless, the relationship between dentition, wearing dental prothesis (including types) and oral problems with frailty and disability has yet to be explored. In this context, the aim of the current study was to examine the relationships of dentition, dental prothesis and oral problems, individually and combined, with frailty and disability in two cohort studies of older adults. Secondly, we have extended our investigations by examining the potential associations of these oral health markers with diet quality.

## Methods

### Data Sources

The current investigation utilised data from the British Regional Heart Study (BRHS) in the UK (28) and the Health, Aging, and Body Composition (HABC) Study in the USA (29). These studies of community-dwelling older adults have complementary data on dentition, dental prothesis, diet, frailty and disability.

The BRHS is an ongoing cohort study with a study population representative of British males, established in 1978–1980, including 7,735 British men (aged 40–59 years) from 24 towns (30). The current analysis used data from the BRHS 40-year follow-up examination in 2018, which was attended by 667 men (41% response rate), while 1009 men (62% response rate) completed a questionnaire (July–December 2018), when aged 78–98 years (31). The physical examination included measures of anthropometry, physical function, and oral health (including more detailed measures on dental prothesis). The questionnaire collected information related to socioeconomic, behavioural and lifestyle factors, current health and medical history, as well as denture status. Ethical approval was provided by the National Research Ethics Service Committee, London. All participants provided written informed consent to participate in the investigations, which were conducted in accordance with the Declaration of Helsinki.

The Health, Aging, and Body Composition (HABC) Study is a prospective cohort study in which 3075 white and African American males and females, aged 70–79 years, were recruited. White participants were selected at random through Medicare, whereas African Americans were selected from neighbourhoods with a zip code around Memphis and Pittsburgh (29). Only individuals who were able to walk 0.25 miles or climb 10 steps without difficulty were included in the study at baseline. In year 2 (1998–1999; n=2998), participants aged 71–80 years completed physical assessments, provided blood samples, completed questionnaires (response rate=97.5%) and a sub-set underwent an oral health assessment (n=1975). All participants provided written informed consent. Ethical approval was provided by University of Pittsburgh, University of Tennessee – Memphis, UCSF and NIH.

### Denture usage and oral health characteristics

In the BRHS, number of teeth and denture usage was assessed by physical examination and by questionnaire; The questionnaire asked, ‘how many of your own (natural) teeth do you have?', whether participants had removable false teeth (full or partial dentures) and, if so, which type or types (partial upper plate, full upper plate, partial lower plate, or full lower plate). There was good agreement between the outcomes of physical examination and self-report (x-index =0.73). In year 2 of the HABC Study, a full mouth assessment was performed by a dental hygienist or a periodontist (32), which included a tooth count and denture usage assessment. In both studies, dental status of participants was based on presence or absence of functional dentition (≥21 teeth), (33) and denture prothesis use and types (34). Dental status was categorized into 5 groups; functional dentition (≥21 teeth) and no dentures; impaired natural dentition (<21 teeth) and no dentures; use of partial denture(s); use of full denture(s); and use of a combination of partial and full denture (33, 34). Questionnaires were also administered in both studies on self-reported oral health measures, including oral pain, difficulty eating, and avoiding foods due to mouth, teeth, or denture problems. Oral health problems were operationalised based on having one or more of these compared to none. To examine the combined effect of dental status and oral health problems, we created ten groups according to both dental status (5-levels) and oral health problems (2-levels; none vs any ≥1) (see Table 1 for details of groupings).

**Table 1:** Ten-level groups for combined measure of dental status with oral problems and prevalence in the BRHS and HABC study

**Group number**	**Definition**	**BRHS (n, %)**	**HABC Study (n,%)**
1	Functional dentition (≥21 teeth) and no dentures without oral problems	206 (28)	688 (35)
2	Functional dentition (≥21 teeth) and no dentures with oral problems	26 (3)	114 (6)
3	Impaired natural dentition (<21 teeth) and no dentures without oral problems	89 (12)	199 (10)
4	Impaired natural dentition (<21 teeth) and no dentures with oral problems	34 (5)	99 (5)
5	Use of partial denture(s) without oral problems	158 (21)	304 (16)
6	Use of partial denture(s) with oral problems	66 (9)	103 (5)
7	Use of full denture(s) without oral problems	71 (10)	190 (10)
8	Use of full denture(s) without oral problems	46 (6)	104 (5)
9	Use of a combination of partial and full denture without oral problems	27 (3)	108 (6)
10	Use of a combination of partial and full denture with oral problems	22 (3)	33 (2)

### Physical frailty, disability, diet quality

In the BRHS and HABC Study, physical frailty was determined based on the Fried frailty phenotype (35), using data from questionnaires and physical assessments at the 40-year and year-2 follow-up, respectively (12, 36). The frailty phenotype includes five components: unintentional weight loss; exhaustion; weakness; low physical activity; and slowness as previously described (18). Participants with none of these components were defined as ‘robust'; with one or two as ‘pre-frail'; and with three or more as ‘frail'. In the BRHS, information on any disability was obtained from the 2018 questionnaire and was based on having mobility limitations (difficulty going up or down stairs or walking 400 yards), activity of daily living (ADL) difficulties (difficulty or needing help doing any of the following tasks: (i) getting in and out of a chair, (ii) dressing and undressing yourself, (iii) bathing or showering, (iv) feeding yourself, including cutting food, or (v) getting to and using the toilet or instrumental (I)ADL problems (any difficulty or needing help in shopping for personal items, preparing your own meals, using telephone by yourself, managing money, or using public transport) (37). In the HABC Study, any disability was defined by any of the following: requiring a cane or walker for ambulation, mobility difficulty (severe difficulty or unable to walk 1/4 mile or climb 10 or more steps) or difficulty with ADL including getting in and out of bed or chairs, bathing/showering, and dressing.

In both studies food frequency (FFQ) questionnaires were completed, the BRHS FFQ was developed for use in the WHO's Monitoring Trends and Determinants in Cardiovascular Disease Survey (38) and the HABC consisted of a 108-item, interviewer-administered modified version of the Block FFQ (Block Dietary Data Systems) (39, 40). In the BRHS, diet quality was calculated as the Elderly Dietary Index (EDI), which is based on the US Modified MyPyramid for Older Adults and other recommendations for older people and comprises of nine food components (meat, fish and seafood, legumes, fruit, vegetables, cereals, bread, olive oil and dairy) (41). In the HABC Study diet quality was assessed by the Healthy Eating Index (HEI) which aligns with the Dietary Guidelines for Americans of 1995 and consists of ten components: nine food components (grains, vegetables, fruit, milk and meat, intakes of percentages of energy content from total and saturated fat, total cholesterol and total Na) and one component which assesses diet variety (39). Further details on both diet scores can be found elsewhere (42).

### Covariates

In both studies, information on sociodemographic factors, behavioural factors (smoking history, and physical activity) and health-related information (i.e., history of cardiovascular disease, diabetes) was available from questionnaires (31, 37). Socio-economic position was based on occupational social class derived from the longest-held occupation when participants entered the study in the BRHS,(43) and according to years of education in the HABC Study (37). In the BRHS, smoking history was defined as current smoker, long-term ex-smoker, recent ex-smoker, and never smoker; alcohol intake was available based on frequency and amount (classified as moderate-heavy and occasional/non-drinkers) drinkers. In the HABC Study, smoking was classified as current, former and never smoker. Physical activity was calculated from questionnaire data on usual time spent in various activities and created as a 2-level category with low activity levels as defined for the frailty phenotype.

### Statistical analysis

All analyses were conducted using SAS v9.4 software (SAS Institute, Inc, Cary, NC) and performed separately for the BRHS and HABC Study. Descriptive characteristics are presented as means and standard deviations for continuous variables and percentages for categorical variables and were compared by dental status by Kruskal-Wallis test and χ2 test, as appropriate.

Separate logistic regression analyses were performed to examine associations of dental status with frailty, and having any disability in the BRHS and HABC Study. The reference group was those with functional dentition. Associations of this combined measure of dental status and oral health problems with both frailty and disability were examined by conducting separate logistic regression models.

Additionally, the association of the combined measure of dental status and oral health problems with diet quality was examined using analysis of covariance (ANCOVA) regression models. The ANCOVA models were used to obtain adjusted means for EDI score in the BRHS, and HEI score in the HABC Study, (42) according to the categories of the combined measure of dental status and oral health problems. Dunnett multiple comparison tests were used for comparing the adjusted means across the groups, with ‘functional dentition and no oral problems' as the reference group (47).

All models were initially adjusted for age as a continuous variable. Model 2 was further adjusted for socioeconomic position, smoking status, moderate/heavy drinking, low physical activity (except for models for frailty), and history of cardiovascular disease (CVD) and diabetes in the BRHS and smoking status, low physical activity (except for models for frailty), history of CVD and diabetes in the HABC study. All covariates were tested for multicollinearity with a tolerance of >0.1 and variance inflation factor <10 before entering into the models.

## Results

### Characteristics of the study populations

In the BRHS, dental status data was available for 855 (80%) British males with a mean age of 85 (±4) years. The majority of the BRHS participants (56%) reported using some type of denture. In the HABC Study, data on number of teeth or denture types was missing for 5 (0.2%) of the 1975 subset who underwent the oral exam. Therefore, the analytical sample comprised of 1970 individuals (50% female) with a mean age of 75 (±3) years. In contrast with the BRHS, the majority of those in the HABC Study were non-denture wearers (57%). The participant characteristics according to dental status in the BRHS and HABC Study are presented in Table 2 and Table 3, respectively. In both studies, a higher proportion of participants with impaired natural dentition (<21 teeth) and not using dentures and those who did use any type of dentures (partial, full or combined) were frail and had disability compared to those with functional dentition and no need for dentures. This was accompanied by a higher proportion of oral health problems including oral pain, self-reported difficulty eating, avoidance of food due to problems with mouth, teeth, or dentures compared with those who had functional dentition and no dentures. According to the 10-level group the highest proportion of individuals had functional dentition (≥21 teeth) and no dentures without oral problems in both studies (Table 1).

**Table 2:** Characteristics of participants in the British Regional Heart Study (BRHS) at the 40-year follow-up (2018) according to dental status

**BRHS**						
**Characteristic**	**No dentures**			**Dentures**		
	**≥21 teeth**	**<21 teeth**	**Partial**	**Full**	**Combined full and partial**	**P value**
	**(n, %; 273, 32)**	**(n, %; 141, 16)**	**(n, %; 245, 29)**	**(n, %; 143, 17)**	**(n, %; 53, 6)**	
Age; mean ± SD	83.7 ± 3	84.8 ± 3.7	84.4 ± 4.3	84.8 ± 4.3	85.0 ± 4.0	<0.01
Social class; n (%)						
Non-manual	193 (71)	78 (55)	148 (60)	59 (41)	31 (58)	<0.001
Manual	80 (29)	63 (45)	97 (40)	84 (59)	22 (42)	
Married; n (%)						
Current smoker; n (%)	3 (2)	1 (1)	6 (3)	1 (2)	2 (5)	0.02
Moderate/heavy drinker; n (%)	9 (3)	4 (3)	8(3)	3 (2)	1 (2)	0.94
Rarely visits dentist; n (%)	7 (3)	12 (9)	4 (2)	41 (35)	5 (9)	<0.001
History of CVD; n (%)	66 (25)	43 (31)	62 (25)	51 (36)	15 (29)	0.12
History of diabetes; n (%)	39 (14)	30 (21)	46 (19)	35 (25)	10 (19)	0.12
Oral health problems; n (%)^a^						
Any None	26 (11.2)	34 (28)	66 (29)	46 (39)	22 (45)	<0.001
Loose fitting dentures; n (%)	N/A	N/A	32 (14)	29 (21)	9 (17)	0.18
Frailty status; n (%)						<0.001
Robust	76 (31)	23 (18)	63 (30)	17 (13)	15 (30)	
Pre-frail	131 (54)	59 (47)	105 (50)	71 (54)	20 (41)	
Frail	38 (15)	43 (35)	43 (20)	44 (33)	14 (29)	
Any disability; n (%)						<0.001
Yes	76 (28)	59 (42)	88 (36)	69 (49)	17 (32)	
No	197 (72)	81 (58)	155 (64)	72 (68)	36 (68)	

Cardiovascular disease (CVD); a. Either oral pain, avoiding foods, or difficulty chewing due to problems with teeth, mouth or dentures; Note: n may vary due to missing data for characteristics.

**Table 3:** Characteristics of participants in the Health Aging and Body Composition (HABC) Study in year 2 (1998–9) according to dental status

**HABC Study**						
**Characteristic**	**No dentures**			**Dentures**		
	**≥21 teeth**	**<21 teeth**	**Partial**	**Full**	**Combined full and partial**	**P value**
	**(n, %; 811, 41)**	**(n, %; 316, 16)**	**(n, %; 407, 21)**	**(n, %; 294, 15)**	**(n, %; 142, 7)**	
Age; mean ± SD	74.7 ± 2.7	74.4 ± 2.9	75.0 ± 3.0	74.7 ± 3.0	74.7 ± 2.8	0.10
Sex; n (%)						0.41
Male	413 (51)	171 (54)	197 (48)	140 (48)	67 (48)	
Female	398 (49)	145 (46)	210 (52)	154 (52)	75 (53)	
Race; n (%)						<0.001
White	632 (78)	136 (43)	245 (60)	124 (42)	73 (51)	
African-American	179 (22)	180 (57)	162 (40)	170 (58)	69 (49)	
Educated less than high school; n (%)	104 (13)	99 (31)	75 (19)	107 (37)	35 (25)	<0.001
Current smoker; n (%)	35 (4.3)	48 (15)	31 (8)	36 (12)	14 (10)	<0.001
Rarely visits dentist; n (%)	127 (15.9)	136 (46)	90 (22)	196 (71)	51 (36)	<0.001
History of CVD; n (%)	190 (23)	65 (21)	106 (26)	94 (32)	47 (33)	<0.01
History of diabetes; n (%)	111 (14)	64 (20)	80 (20)	82 (28)	39 (28)	<0.001
Oral health problems; n (%)^a^	114 (14)	99 (33)	103 (25)	104 (35)	33 (23)	<0.001
Frailty status; n (%)						
Robust	420 (53)	114 (39)	213 (54)	115 (40)	68 (49)	<0.001
Pre-frail	329 (42)	158 (54)	171 (43)	156 (55)	63 (45)	
Frail	38 (5)	21 (7)	14 (3)	15 (5)	9 (6)	
Any disability; n (%)						<0.001
Yes	144 (18)	81 (26)	95 (23)	85 (29)	29 (21)	
No	666 (82)	235 (74)	311 (77)	209 (71)	112 (79)	

Cardiovasular disease (CVD); a. Either oral pain, avoiding foods, or difficulty chewing due to problems with teeth, mouth or dentures

Associations of dental status and oral health problems with frailty and disability

Odds ratios (OR) and 95% CI for the separate associations of dental status and oral health problems with frailty and disability in the BRHS and HABC Study are presented in Table 4. In the BRHS, age-adjusted models for having impaired natural dentition and no dentures, full denture(s) compared to functional dentition and no dentures showed a higher likelihood of frailty and disability. Associations of impaired natural dentition and no dentures and full denture(s) with frailty remained significant after full adjustment. In the HABC Study, age-adjusted associations were observed for impaired natural dentition and no dentures, partial and full denture(s) compared to functional dentition and no dentures with disability. Dental status was no longer associated with disability in the multivariate analysis after full adjustment.

**Table 4:** Cross-sectional associations (odds ratio (95% confidence interval)) of dental status and oral health problems with frailty and disability in the BRHS and HABC Study

	**Frailty**		**Any disability**	
	**Age-adjusted**	**Model 2**	**Age-adjusted**	**Model 2**
**BRHS (2018)**				
*Dental status*				
Functional dentition (≥21 teeth) and no dentures	1.00	1.00	1.00	1.00
Impaired natural dentition (<21 teeth) and no dentures	**2.47 (1.47, 4.17)**	**2.32 (1.33, 4.03)**	**1.70 (1.10, 2.63)**	1.47 (0.89, 2.45)
Partial denture(s)	1.24 (0.76, 2.04)	1.16 (0.68, 1.97)	1.37 (0.94, 2.00)	1.43 (0.93, 2.22)
Full dentures(s)	**2.31 (1.37, 3.89)**	1.90 (1.07, 3.40)	**2.28 (1.48, 3.51)**	1.58 (0.93, 2.67)
Combination of full and partial dentures	1.80 (0.86, 3.76)	1.52 (0.69, 3.34)	1.06 (0.55, 2.01)	0.71 (0.34, 1.52)
*Oral health problems*^*a*^				
None	1.00	1.00	1.00	1.00
Any	**2.01 (1.39, 2.90)**	**1.75 (1.17, 2.60)**	**1.06 (1.50, 2.84)**	**1.76 (1.20, 2.59)**
**HABC Study (1998–9)**				
*Dental status*				
Functional dentition (≥21 teeth) and no dentures	1.00	1.00	1.00	1.00
Impaired natural dentition (<21 teeth) and no dentures	1.57 (0.90, 2.73)	1.17 (0.65, 2.12)	**1.62 (1.18, 2.20)**	1.35 (0.96, 1.92)
Partial denture(s)	0.70 (0.37, 1.29)	0.54 (0.28, 1.02)	**1.39 (1.04, 1.86)**	1.29 (0.95, 1.76)
Full dentures(s)	1.07 (0.58, 1.98)	0.60 (0.31, 1.19)	**1.88 (1.38, 2.56)**	1.35 (0.96, 1.92)
Combination of full and partial dentures	1.24 (0.63, 2.84)	0.66 (0.28, 1.56)	1.19 (0.76, 1.87)	0.85 (0.52, 1.38)
*Oral health problems*^*b*^				
None	1.00	1.00	1.00	1.00
Any	**1.85 (1.32, 2.61)**	**1.56 (1.09, 2.22)**	**1.73 (1.36, 2.20)**	**1.54 (1.19, 1.99)**

Model 2 further adjusted for social class, smoking status, moderate/heavy drinking, low physical activity (except for model for frailty), history of cardiovascular disease and diabetes in BRHS and for sex, race, level of education, smoking status, low physical activity (except frailty), history of cardiovascular disease and diabetes in HABC Study; a. Oral pain, avoiding foods, or difficulty chewing due to problems with teeth, mouth or dentures; b. Oral pain, avoiding foods, or difficulty chewing due to problems with teeth, mouth or dentures. Bold represents significance P<0.05.

In both studies, a relationship was observed between oral problems related to eating with frailty and disability. In the multivariate analysis, those with oral health problems were ∼1.8 and ∼1.5 times more likely to be frail or have disability compared to those without in the BRHS and HABC Study, respectively.

### Combined associations of dental status and oral health problems with frailty, disability and diet quality

A combined measure including both dental status and oral health problems was also examined (Table 1). Full results for the associations of the combined measure of dental status and oral health problems (functional dentition (≥21 teeth) and no dentures without oral problems as the reference group) with frailty and disability in the BRHS and HABC Study are presented in Table 5.

**Table 5:** Cross-sectional associations (odds ratio (95% confidence interval)) of combined measure of dental status and oral health problems^1^ with frailty and disability in the BRHS and HABC Study

**BRHS (2018)**						
		**Frailty**			**Any disability**	
	**n (%)**	**Age-adjusted**	**Model 2**	**n (%)**	**Age-adjusted**	**Model 2**
*Dental status and oral problems*						
Group 1 (ref)	26 (14)	1.00	1.00	55 (27)	1.00	1.00
Group 2	7 (28)	2.09 (0.78, 5.61)	1.77 (0.60, 5.17)	10 (38)	2.09 (0.78, 5.61)	1.33 (0.48, 3.71)
Group 3	21 (27)	**2.08 (1.07, 4.07)**	1.95 (0.96, 3.93)	31 (35)	**2.08 (1.07, 4.07)**	1.33 (0.71, 2.49)
Group 4	14 (44)	**3.83 (1.65, 8.92)**	**3.24 (1.30, 8.03)**	18 (55)	**3.83 (1.65, 8.92)**	2.31 (0.91, 5.87)
Group 5	27 (20)	1.34 (0.73, 2.46)	1.26 (0.66, 2.39)	52 (33)	1.34 (0.73, 2.46)	1.37 (0.80, 2.33)
Group 6	12 (21)	1.51 (0.69, 3.30)	1.35 (0.59, 3.10)	28 (42)	1.52 (0.69, 3.30)	**2.03 (1.03, 4.00)**
Group 7	16 (25)	1.84 (0.89, 3.79)	1.37 (0.61, 3.04)	28 (39)	1.84 (0.89, 3.79)	1.19 (0.58, 2.44)
Group 8	20 (45)	**4.02 (1.87, 8.64)**	**3.67 (1.61, 8.34)**	30 (65)	**4.01 (1.87, 8.64)**	**3.20 (1.42, 7.19)**
Group 9	7 (28)	2.04 (0.75, 5.54)	1.91 (0.68, 5.33)	8 (30)	2.04 (0.75, 5.54)	0.69 (0.26, 1.88)
Group 10	6 (30)	2.02 (0.68, 6.01)	1.34 (0.40, 4.48)	9 (41)	2.02 (0.68, 6.01)	1.17 (0.37, 3.69)
**HABC Study (1998–9)**						
		**Frailty**			**Any disability**	
	**n (%)**	**Age-adjusted**	**Model 2**	**n (%)**	**Age-adjusted**	**Model 2**
*Dental status and oral problems*						
Group 1 (ref)	26 (4)	1.00	1.00	118 (17)	1.00	1.00
Group 2	12 (11)	**3.03 (1.48, 6.22)**	**3.01 (1.44, 6.31)**	24 (21)	1.29 (0.79, 2.10)	1.28 (0.77, 2.13)
Group 3	12 (7)	1.72 (0.85, 3.49)	1.38 (0.66, 2.88)	46 (23)	**1.48 (1.01, 2.17)**	1.32 (0.88, 1.99)
Group 4	8 (8)	2.24 (0.98, 5.11)	1.56 (0.65, 3.78)	29 (30)	**2.01 (1.25, 3.24)**	1.63 (0.97, 2.74)
Group 5	8 (3)	0.65 (0.29, 1.46)	0.51 (0.22, 1.17)	60 (20)	1.16 (0.82, 1.64)	1.09 (0.76, 1.57)
Group 6	6 (6)	1.59 (0.64, 3.98)	1.24 (0.49, 3.19)	35 (34)	**2.49 (1.58, 3.93)**	**2.28 (1.41, 3.69)**
Group 7	9 (5)	1.22 (0.56, 2.66)	0.64 (0.27, 1.51)	47 (25)	**1.57 (1.07, 2.31)**	1.19 (0.79, 1.81)
Group 8	6 (6)	1.63 (0.65, 4.08)	1.06 (0.40, 2.80)	38 (37)	**2.80 (1.79, 4.38)**	**1.91 (1.17, 3.13)**
Group 9	6 (6)	1.51 (0.60, 3.76)	1.00 (0.39, 2.57)	21 (19)	1.17 (0.70, 1.97)	0.88 (0.51, 1.51)
Group 10	3 (9)	2.29 (0.65, 8.03)	0.41 (0.05, 1.44)	8 (24)	1.49 (0.65, 3.38)	0.92 (0.35, 2.39)

In the BRHS age-adjusted associations were found for impaired natural dentition (<21 teeth) and no dentures with oral problems and use of full denture(s) without oral problems with both frailty and disability. Increased likelihood of frailty remained significant in these groups in the multivariate analysis. However, in the multivariate model the relationship between impaired natural dentition (<21 teeth) and no dentures and disability was no longer significant. In the final model prevalence of disability was higher in those who used partial denture(s) or full denture(s) that had oral problems.

In the HABC Study an age-adjusted association for functional dentition (≥21 teeth) and no dentures with oral problems and frailty was found. The likelihood of frailty was higher in this group even after further adjustment. Univariate associations were found for impaired natural dentition (<21 teeth) and use of full denture(s) with disability, regardless of oral problems. Those with partial denture(s) and oral problems were also more likely to have disability. In the multivariate model only those with partial or full denture(s) and oral problems remained significant.

**Figure 1:**
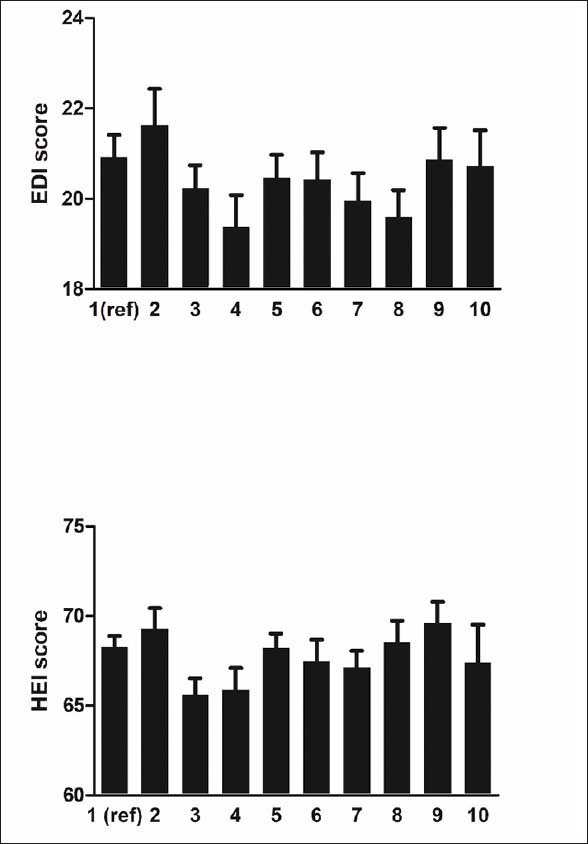
Means score of diet quality according to combined measure of dental status and oral health in the BRHS (top figure – elderly diet index; EDI) and HABC Study (bottom figure –healthy eating index; HEI) Adjusted for age, social class, smoking status, moderate/heavy drinking, low physical activity, history of cardiovascular disease and diabetes in the BRHS and age, sex, race, level of education, smoking status, low physical activity, history of cardiovascular disease and diabetes in the HABC Study. *P<0.05

The joint associations for dental status and oral problems with diet quality are shown in Figure 1. There were no significant differences found between diet quality compared to those with functional dentition and no oral problems after controlling for sociodemographic, behavioural and health factors.

## Discussion

To the best of our knowledge this is the first study to investigate the associations of dentition and denture types, independently and combined with oral problems, with frailty and disability. The main finding of this study was that in two studies of older adults in the UK and USA, oral problems related to eating and dental status with the presence of oral problems were associated with higher odds of frailty and disability even after adjusting for important sociodemographic, behavioural and health factors.

This study has several strengths. We examined dental status in two comparable, but independent, studies of community-dwelling older people in two geographically different Western populations. The HABC Study has specifically been designed to look at decline in function in older adults, while the BRHS has updated measures to include physical function in the latest follow-up of older adults. Moreover, the detailed measures available in both studies allowed us to adjust for important confounders. Nevertheless, we recognise several limitations of this study. Firstly, our findings were cross-sectional, and therefore cannot establish a causal relationship. Secondly, design features of and the populations of both studies limit the generalizability of our findings. Thirdly, the different measures used for some outcomes in the two studies (e.g., for disability and diet quality), might account for differences in results between the two studies. Fourth, the numbers in the joint associations were lower and this resulted in wide confidence levels in the associations, but the commonality of results between studies for disability adds confidence to these findings. Lastly, the results should be interpreted with caution given: (i) we cannot exclude the possibility of residual confounding variables, (ii) there is a potential risk of attrition bias (iii) we undertook multiple comparisons which increases the risk of false-positive results, and (iv) we did not have information on aspects such as quality or functionality of dentures in our analyses.

With regards to dental status, the findings of the current study are consistent with our previous findings that there was a greater burden of frailty among non-denture wearers with impaired natural dentition compared with those with functional dentition who did not need dentures (18). Interestingly in the same study in the BRHS, even those who wore dentures were still more likely to progress to frailty, however we were not able to differentiate by denture types due to limited data on dentures collected at the 30-year follow-up. In the most recent BRHS follow-up examination in 2018 more detailed information on dentures was collected (31) and hence has been included in this study when the participants were much older (∼10 years) than previous reports (13, 18, 24, 44). After controlling for important confounders, we found no association between denture types with frailty or disability compared to those with functional dentition. The relationship of denture types with frailty and disability has received limited attention. Our findings contrast that of Everaars et al. (23) who reported that wearing a partial or full prothesis was associated with 1.5 and 3.3 higher likelihood of frailty compared with those with natural dentition, respectively. This contradiction may be related to the differences in frailty scoring tools between ours and their study, i.e., Frailty Index. However, in another study in that types of dentures were not associated with malnutrition, which includes weight loss a component of frailty, in older adults (34). Another potential explanation for these disparities in findings is that, in some instances, dentures may improve oral health (45), while in other instances wearing dentures are related to problems with retention, stability, ill-fitting dentures, reduced masticatory function and efficiency, and in turn difficulty in eating (20, 21, 46, 47). In support of this we found in line with others that oral health problems irrespective of dental status, are associated with frailty and disability, falls, hospitalisation and mortality in older adults (48).

In the current study we conducted a combination analysis demonstrating that dental status along with oral health problems related to eating were jointly associated with frailty and disability. Of note, in both studies participants wearing full or partial denture(s) who reported oral problems related to eating had higher odds of disability even after controlling for important confounders, while the associations with dental status alone were attenuated. The same groups were also more likely to be frail in the BRHS study. Accordingly, among denture wearers, previous studies have suggested those with problems such as difficulty in chewing/swallowing or ill-fitting dentures are more likely to become weak or frail (26, 49). A strength of our study compared to those is that we were able to differentiate by denture types. As this is the first study to look at the relationship between dental status combined with oral problems with disability it makes it hard to compare to previous literature. Although a limitation remains that we only used self-reported measures of oral problems rather than denture quality or functionality. Nonetheless, our findings from both studies support that self-reported oral problems related to eating (which are relatively simple to assess), rather than dental status alone, may be more informative predictors of frailty and disability in older adults. Particularly since in the HABC Study even those with functional dentition and no need for dentures with oral problems and in the BRHS impaired natural dentition (<21 teeth) and no dentures with stratified by oral problems had a higher probability of being frail.

One potential mechanism mediating these associations with frailty and disability is the impact of oral health on nutrition. It is well established that having less than 21 teeth, generally considered the minimum to maintain adequate oral function, is associated with avoiding a number of foods that are hard to chew, such as fruits, vegetables, meat and nuts, that are components of healthy dietary patterns (50, 51). However, whilst dentures may improve oral function, use of dentures (partial or full) can result in compromised nutritional intake and diet quality (20). Together with oral health, diet is an important modifiable risk factor for frailty and disability, making it a potential mediating factor (17, 25, 36, 44, 52, 53). For example, in two recent studies, non-functional dentition was associated with musculoskeletal frailty and ADL problems and nutritional intake and eating difficulties, respectively, were shown to partially explain these associations (17, 54). While we were not able to show a difference in diet among dental status combined with oral health problems in the current study, we and others have reported limited associations between oral health and diet quality scores (42, 55) which might be explained by the limitations of the tools used to assess diet quality In addition, besides impacts on nutritional status, oral health can impact communication, aesthetics and social activity which could in turn contribute to disability (56, 57). For example, tooth loss has been associated with IADL problems in older adults, and 22% of this association was explained by self-reported communication difficulties, while eating difficulty was not a significant mediator (54). Furthermore, mastication can influence cognitive function via alternate ways to nutrition such as by increasing blood flow and neuronal activities; and cognitive impairment is associated with frailty, disability, and oral health in a bidirectional manner (58, 59, 60). Therefore, the relationship between oral health and comorbidities in older adults is likely complex and disentangling the causal relationships is challenging. Nonetheless, the current cross-sectional study demonstrates that oral health problems related to eating are a potential modifier of the relationship between dental status with frailty and disability and that these simple measures may be indicators of frailty and disability in later life.

Future studies should investigate the prospective associations between dental status and frailty/disability, the possible mediating effects of change in diet and eating habits. Research would benefit from utilising more robust measures of dietary intake, including objective biomarkers (61), to help clarify diet as a potential pathway. In addition, studies should assess the objective quality and functionality of dentures, rather than self-reported measures, to establish whether this might help identify those at risk of frailty and disability. If confirmed in further studies, this suggests that healthcare professionals should consider including such questions in both individual patient care and in surveys of health in older people.

In summary, our findings, and the those of others, suggest that impaired natural dentition without using a denture and oral problems are associated with frailty. In addition, simple self-reported oral health problems related to eating may modify the relationship between dental status with frailty and disability. These data suggest dental status together with simple self-reported oral health problems related to eating could help identify those at risk of frailty and disability in older age.

## Author contributions

Study concept and design: RK, SER, SGW, AOP, PHW, JCM, RJW and LTL. Acquisition of data: SER, SGW, AOP, PHW, RJW and LTL. Analysis and interpretation of data: All authors. Drafting of the manuscript: All authors. Critical revision of the manuscript for important intellectual content: All authors.

## Funding

The research was supported by core funding from the British Heart Foundation [since 2009 this has included both programme grants (RG/08/013/25942, RG/13/16/30528, RG/19/4/34452) and project grants (PG/13/86/30546 and PG/13/41/30304)] and National Institute on Aging (NIA) contracts #N01-AG-6-2101; N01-AG-6-2103; N01-AG-6-2106; NIA grant (R01-AG028050); NINR grant (R01-NR012459). Funding has also been received from the Medical Research Council (G1002391) Dunhill Medical Trust (R592_0717, R592_0515 and R396_1114) and US NIH/NIDCR grant R03 DE028505-02. SER is a member and investigator of Fuse, the Centre for Translational Research in Public Health (www.fuse.ac.uk). Fuse is a UK Clinical Research Collaboration (UKCRC) Public Health Research Centre of Excellence. Funding for Fuse from the British Heart Foundation, Cancer Research UK, National Institute for Health Research, Economic and Social Research Council, Medical Research Council, Health and Social Care Research and Development Office, Northern Ireland, National Institute for Social Care and Health Research (Welsh Assembly Government) and the Wellcome Trust, under the auspices of the UKCRC, is gratefully acknowledged.

## Conflicts of interest

The Authors declare no conflicts of interest.
